# Schadensaggregation in der Rückversicherung – die Principles of Reinsurance Contract Law (PRICL) als Mittel zur Schaffung höherer Rechtssicherheit?

**DOI:** 10.1007/s12297-020-00476-8

**Published:** 2020-10-06

**Authors:** Oliver D. William

**Affiliations:** 1 Universität Bern, Bern, Schweiz; 2 Rechtsanwalt mbh ATTORNEYS AT LAW, Zürich, Schweiz

## Abstract

Kaum eine Rechtsordnung kennt Regeln für die Schadenaggregation in der Rückversicherung. Eine Ausnahme bietet das englische Recht, wo sich diesbezüglich eine reiche Rechtsprechung entwickelt hat.

Eine Analyse dieser Rechtsprechung zeigt, dass im Bereich der Schadenaggregation in der Rückversicherung terminologische Unsicherheiten bestehen und dass die von der Rechtsprechung entwickelten Beurteilungsmassstäbe nicht geeignet sind, Rechtssicherheit herzustellen. Dieser Zustand wird der Bedeutung der nichtproportionalen Rückversicherung nicht gerecht.

Mit dem Ziel, den Rückversicherungsmärkten mehr Rechtssicherheit zu bieten, schlägt Kapitel 5 der Principles of Reinsurance Contract Law (PRICL) neu konzipierte Regeln zur Schadenaggregation vor.

## Abstract (en)

Most legal systems do not contain any rules regarding the aggregation of losses in the field of reinsurance. As an exception, the law of England and Wales has developed a jurisprudential riches on the subject matter.

An analysis of the case law shows that the English law of aggregation is characterized by considerable terminological uncertainties and that the tests and standards developed by English courts are inadequate for creating legal certainty in the matter. Considering the importance of nonproportional reinsurance, this situation of legal uncertainty is unbearable.

With a view to creating more legal certainty in the area, chapter 5 of the Principles of Reinsurance Contract Law (PRICL) provides for newly designed rules on the aggregation of losses in reinsurance.

## Ausgangslage

Die nichtproportionale Rückversicherung (*excess of loss reinsurance*) zeichnet sich dadurch aus, dass der Rückversicherer lediglich jenen Teil eines Schadens trägt, der über die Priorität (*retention*) des Zedenten hinausgeht, das Deckungslimit (*cover limit*) jedoch nicht übersteigt.^1^ Priorität und Deckungslimit prägen den quantitativen Deckungsumfang nichtproportionaler Rückversicherungen damit massgeblich.^2^ Die Überprüfung, ob ein Schaden Priorität bzw. Deckungslimit übersteigt, setzt allerdings ein klares Verständnis dessen voraus, was als *ein* Schaden anzusehen ist.

In sog. Einzel- und Kumulschadenexzedentenrückversicherungen^3^ sehen die Parteien regelmässig Aggregationsklauseln vor, nach welchen all jene Einzelschäden, die durch ein bestimmtes Merkmal miteinander verbunden sind, mit Blick auf Priorität und Deckungslimit zusammen als *ein* Schaden gelten. Mit anderen Worten werden diese Einzelschäden aggregiert. Nur wenn das Aggregat die Priorität übersteigt, entsteht beim Rückversicherer eine Leistungspflicht.

In der deutschsprachigen Literatur werden Aggregationsklauseln oftmals als „Serienschadenklauseln“ bezeichnet.^4^ Gemäss dem Gabler Versicherungslexikon sind „Serienschadenklauseln“ Vereinbarungen in Versicherungsverträgen, nach denen „mehrere zeitlich zusammenhängende Schäden aus derselben Ursache als ein Schadenereignis gelten“.^5^ Nach englischem Verständnis bilden sog. „*claims series clauses*“ jedoch nur eine Unterkategorie der Aggregationsklauseln. Dabei handelt es sich nämlich um Aggregationsklauseln, die explizit auf das Verbindungsmerkmal „*series*“ abstellen.^6^

Aggregationsklauseln sind i. d. R. sehr abstrakt gehalten und weisen ein nicht zu unterschätzendes Mass an Komplexität auf. So sind sich die Parteien über deren Auslegung oftmals uneinig.^7^ Trotzdem hatte – soweit ersichtlich – weder die deutsche^8^ noch die schweizerische Judikatur bisher Gelegenheit, sich mit dem Thema der Schadenaggregation in der Rückversicherung auseinanderzusetzen.^9^ Es erscheint daher sinnvoll, die reiche englische Rechtsprechung zur Auslegung von Aggregationsklauseln auch für Rückversicherungsverträge zu berücksichtigen, die deutschem oder schweizerischem Recht unterstehen.^10^ Dies muss umso mehr gelten, als die Rückversicherungsindustrie äusserst international agiert^11^ und davon auszugehen ist, dass Parteien Aggregationsmechanismen relativ unabhängig vom anwendbaren Recht definieren.^12^ Das schweizerische Bundesgericht erklärte jedenfalls ausdrücklich, dass die von Entscheidungen englischer Gerichte geprägte Rückversicherungspraxis für das schweizerische Rückversicherungsvertragsrecht bedeutsam sei.^13^ Auch in der spärlichen deutschen Literatur zum Thema der Schadenaggregation in der Rückversicherung wird auf die englische Rechtsprechung Bezug genommen.^14^ Im Folgenden wird entsprechend vornehmlich englisches Recht untersucht und es soll nicht von „Serienschadenklauseln“, sondern von „Aggregationsklauseln“ gesprochen werden.

## Bedeutung einer rechtssicheren Schadenaggregation

Die vom Zedenten dem Rückversicherer als Gegenleistung für die Übernahme des Risikos zu bezahlende Rückversicherungsprämie wird anhand der Wahrscheinlichkeit berechnet, dass Schäden eintreten, welche die Priorität übersteigen. Dabei spielen u. a. auch die erwartete Frequenz solcher Schäden und deren Höhe eine Rolle.^15^ Ohne Verständnis eines Aggregationsmechanismus ist es nicht möglich, diese Wahrscheinlichkeit, Frequenz und Höhe zu berechnen. Aus diesem Grund erweisen sich Aggregationsmechanismen für die Prämiengestaltung als äusserst relevant.^16^

Weiter setzt eine risikoadäquate Kapitalisierung eines Versicherungsträgers eine gewisse ökonomische Werthaltigkeit des Versicherungsschutzes^17^ und damit unter anderem auch Rechtssicherheit über die Funktionsweise eines Aggregationsmechanismus voraus.^18^ Entsprechend erscheinen Aggregationsmechanismen auch aus aufsichtsrechtlicher Perspektive als bedeutsam.

Aggregationsklauseln sind in vielen verschiedenen Versicherungszweigen im Bereich der Erst- und Rückversicherung üblich. Aus aktuellem Anlass wird z. B. diskutiert, wie Aggregationsklauseln in Betriebsschliessungsversicherungen mit Blick auf die Covid-19 Pandemie auszulegen sind.^19^ Ein weiteres Feld, in dem Aggregationsklauseln als höchst unsicher gelten, ist die Cyberversicherung.^20^

Um mit Lord Mustills Worten zu sprechen, sind Aggregationsklauseln in nichtproportionalen Rückversicherungsverträgen „of cardinal importance“.^21^ Seit 1995 wurden vor englischen Gerichten vermehrt Streitigkeiten über die Schadenaggregation ausgefochten.^22^ O’Neill, Woloniecki und Arnold-Dwyer sprechen von einem „embarrasment of jurisprudential riches“,^201^ das bisher kaum zu Rechtssicherheit beigetragen hat.^23^ Für ein effizientes Geschäft ist diese Unsicherheit allerdings alles andere als zuträglich.^24^ Entsprechend hält Barlow Lyde & Gilbert LLP die Schadenaggregation für „one of the most vexing issues facing the reinsurance market in recent years“.^25^

Im Folgenden soll zunächst auf den Einzelschaden als Grundbaustein eines Schadenaggregats eingegangen werden (Abschn. 3). Weiter wird der Geltungsgrund eines Aggregationsmechanismus sowie dessen Grundkonzept erläutert (Abschn. 4), bevor auf die verbreitete ereignisbasierte (*event-based*) (Abschn. 5) bzw. ursachenbasierte (*cause-based*) (Abschn. 6) Schadenaggregation einzugehen ist. Sodann sollen die Regelungen der Principles of Reinsurance Contract Law (PRICL) zur ereignisbasierten bzw. ursachenbasierten Schadenaggregation überblicksartig vorgestellt werden (Abschn. 7). Schliesslich werden die Ergebnisse in einem Fazit zusammengefasst (Abschn. 8).

## Was ist ein Einzelschaden?

Wie bereits erwähnt, erlauben Aggregationsklauseln, dass mehrere Einzelschäden, die durch ein bestimmtes Merkmal miteinander verbunden sind, mit Blick auf Priorität und Deckungslimit zusammengezählt werden. In Aggregationsklauseln werden Einzelschäden als „*losses*“, „*claims*“ oder gelegentlich „*occurrences*“ bezeichnet.^26^ Eine Aufarbeitung der englischen Rechtsprechung zur Verwendung dieser Begriffe in Aggregationsklauseln ergibt, dass keineswegs klar ist, ob sie synonym verwendet werden oder inhaltliche Unterschiede aufweisen.^27^

Der Begriff „*claims*“ wird regelmässig – aber nicht immer – verwendet, wenn Haftpflichtrisiken rückversichert werden.^28^ Die Bezeichnung von Einzelschäden als „*occurrences*“ hat in der Branche zu Missverständnissen geführt.^29^ Denn das Wort „*occurrence*“ wird in Aggregationsklauseln in der Regel nicht zur Bezeichnung eines Einzelschadens, sondern vielmehr zur Bezeichnung eines Verbindungsmerkmals (*unifying factor*) verwendet.^30^ In *Caudle v Sharp* hielt Lord Justice Evans im Court of Appeal etwa fest, „[t]he relevant occurrence, strictly, is the making of a claim or the discovery of a loss by the original insured, *but the word more readily means the occurrence out of which a claim arises* […].“^31^ In diesem Sinne wird der Begriff „*occurrence*“ denn auch immer wieder mit dem Ausdruck „*event*“ bzw. „*Ereignis*“ gleichgesetzt.^32^ Trotzdem ist es ratsam, im Einzelfall zu prüfen, ob das Wort „*occurrence*“ im Sinne eines Einzelschadens oder im Sinne eines Ereignisses verwendet wird.^33^

Neben diesen terminologischen Unsicherheiten besteht im englischen Recht auch inhaltlich keine Klarheit darüber, wie Einzelschäden voneinander abzugrenzen sind.^34^ Fraglich kann z. B. sein, ob ein Erdbeben ein oder zwei Einzelschäden verursacht, wenn es zwei verschiedene sich auf demselben Grundstück befindliche Gebäude beschädigt. Ebenso unsicher ist, ob einer Fluggesellschaft ein Einzelschaden oder mehrere Einzelschäden entsteht bzw. entstehen, wenn Entführer gleichzeitig mehrere Flugzeuge entführen.^35^

Zur Bestimmung, was ein Einzelschaden ist, werden im englischen Recht mehrere verschiedene Ansätze verfolgt: gelegentlich wird darauf abgestellt, dass in einer Versicherungspolice mehrere versicherte Objekte aufgelistet sind. Für jedes Objekt, das Schaden nimmt, wird ein separater Einzelschaden angenommen. In *Kuwait Airways Corp v Kuwait Insurance Co SAK* waren 23 Flugzeuge der Kuwait Airways unter einer einzigen Police versichert. Im Zuge der Invasion von Kuwait durch den Irak im Jahre 1990 wurden 15 der 23 Flugzeuge durch irakische Militärs in Gewahrsam genommen und in den Irak geflogen. Weil die Flugzeuge in der Police als separate versicherte Objekte aufgeführt waren, ging man von 15 Einzelschäden aus.^36^ Oftmals sind versicherte Objekte in einer Versicherungspolice allerdings nicht fein säuberlich aufgeschlüsselt, sodass die Frage, was ein Einzelschaden ist, zur anspruchsvollen Auslegungsfrage wird.^37^

Nach einem anderen Ansatz werden Handlungen und Unterlassungen von Personen, welche die Schäden verursacht haben, zum Massstab für die Beurteilung der Anzahl entstandener Einzelschäden genommen.^38^ So erkannten englische Gerichte, dass im Rahmen einer „*occassion in the course of one enterprise*“ jeweils nur ein Einzelschaden entstehen könne.^39^ Demgegenüber sind Schäden, die durch mehrere unabhängige Handlungen verursacht werden, als mehrere Einzelschäden zu beurteilen.^40^ Wenn aus einer Handlung aber nur *ein* Einzelschaden hervorgehen kann, so stellt sich die Frage der Schadenaggregation in Bezug auf diese Handlung gar nicht.^41^

Es erscheint ungeklärt, wann welcher Ansatz zum Tragen kommt. Englische Gerichte scheinen in dieser Frage eher einzelfallabhängig vorzugehen. Ein übergeordnetes Konzept mit einer Rangordnung der verschiedenen Vorgehensweisen zur Abgrenzung von Einzelschäden scheint nicht zu existieren. Unter englischem Recht besteht entsprechend eine erhebliche Unsicherheit darüber, was überhaupt als Einzelschaden gilt.

Einig scheint man sich demgegenüber darüber zu sein, dass die Einzelschäden dem Versicherungsnehmer im Erstversicherungsverhältnis anfallen und nicht in jedem Fall mit den Schäden des Zedenten übereinstimmen, die diesem aufgrund seiner Leistungspflicht im Erstversicherungsverhältnis entstehen.^42^

## Vertraglicher Geltungsgrund und Kausalität als Grundkonzept der Schadenaggregation in Einzelschaden- und Kumulschadenexzedenten

Im Gegensatz zum englischen Recht^43^, entspricht es zwar dem deutschen Verständnis, dass ein Zedent grundsätzlich eine Priorität bzw. einen Selbstbehalt zu tragen hat.^44^ Abgesehen davon, steht es aber in der Disposition der Parteien, wie sie den quantitativen Deckungsumfang ihres Rückversicherungsvertrags ausgestalten.^45^ Mit anderen Worten steht es den Parteien frei, einen Einzelschaden oder aber ein Aggregat von Einzelschäden an Priorität und Deckungslimit zu messen. In Ermangelung einer expliziten Aggregationsklausel sind Einzelschäden nach englischem Recht nicht zu aggregieren. Eine richterliche Ergänzung des Rückversicherungsvertrags um eine Aggregationsklausel kommt grundsätzlich jedenfalls nicht in Betracht.^46^

In diesem Sinne steht auch die Ausgestaltung eines Aggregationsmechanismus im Belieben der Parteien.^47^ So können die Parteien z. B. vereinbaren, dass all jene Einzelschäden, die während derselben Rückversicherungsperiode anfallen, zu aggregieren sind (sog. Jahresüberschadenrückversicherung; *aggregate excess of loss reinsurance*).^48^ In diesem Fall bedürfen die Einzelschäden zur Aggregation keines gemeinsamen Ursprungs.^49^ Demgegenüber wird in sog. Schadenexzedentenrückversicherungen (*excess of loss reinsurance*) vereinbart, dass Einzelschäden nur dann aggregiert werden, wenn sie ein in der Aggregationsklausel designiertes Verbindungsmerkmal (*unifying factor*) aufweisen. Der englische Court of Appeal spricht in diesem Zusammenhang von „*related*“ *losses* oder *claims*.^50^ Im Kern solcher Aggregationsmechanismen liegt ein Kausalitätserfordernis zwischen dem Verbindungsmerkmal und den Einzelschäden.^51^

Verschiedene Aggregationsmechanismen unterscheiden sich in ihren unterschiedlichen Kausalitätsanforderungen. Sprachlich finden diese Anforderungen in der Nennung von Verbindungsmerkmal (*unifying factor*) und Verbindungsworten (*linking phrase*)^52^ Eingang in Aggregationsklauseln.^53^ Als *unifying factors* werden z. B. Ereignisse (*events; occurrences*^54^), Ursachen (*originating/original causes*), Unfälle (*accidents*), Handlungen und Unterlassungen von Schädigern (*acts and omissions*) und Naturkatastrophen (*catastrophes; disasters; calamities*) verwendet.^55^ Als *linking phrases* werden Ausdrücke bezeichnet, welche die Verbindung zwischen den Einzelschäden und dem relevanten *unifying factor* herstellen.^56^ Übliche *linking phrases* sind etwa „*arising out of*“, „*arising from*“, „*consequent upon or attributable to*“, „*in connection with*“ und „*shall result from*“.^57^

Englische Gerichte beschäftigen sich in erster Linie mit der Wortauslegung der in Aggregationsklauseln verwendeten Begriffe im Einzelfall.^58^ Am Beispiel zweier viel verwendeter Kombinationen von *unifying factor* und *linking phrase* soll im Anschluss gezeigt werden, dass die Bestimmung der Kausalitätserfordernisse eines Aggregationsmechanismus und deren Beurteilung im Einzelfall oftmals alles andere als eindeutig ausfallen.

## Ereignisbasierte Schadenaggregierung (*event-based aggregation*)

Eine typische ereignisbasierte Aggregationsklausel lautet etwa: „For the purpose of this reinsurance the term ‚each and every loss‘ shall be understood to mean each and every loss […] arising out of one event“.^59^ Die Klausel verlangt, dass all jene Einzelschäden aggregiert werden, die auf dasselbe Ereignis (*event*) zurückzuführen sind. Das Ereignis fungiert als *unifying factor*. Die Kausalitätserfordernisse werden zudem vom *linking phrase* „*arising out of*“ mitgeprägt.

### Ereignisbegriff und Kausalitätsanforderungen

In *Caudle v Sharp* legte der englische Court of Appeal eine ereignisbasierte Aggregationsklausel aus. Lord Justice Evans erklärte, der Aggregationsmechanismus würde sich aus drei Elementen zusammensetzen. Zunächst müsse etwas passiert sein, das die Einzelschäden verbinde und als Ereignis bezeichnet werden könne; zweitens müsse zwischen diesem Ereignis und jedem Einzelschaden ein Kausalzusammenhang bestehen, der, drittens, nicht zu schwach sein dürfe.^60^

Der Begriff des Ereignisses (*event*) wird in der Rückversicherung in verschiedenen Zusammenhängen und entsprechend mit unterschiedlichen Bedeutungen gebraucht.^61^ In *Axa Reinsurance (UK) Ltd v Field* definierte Lord Mustill den Ereignisbegriff im Zusammenhang mit Aggregationsklauseln folgendermassen: ein Ereignis ist „something which happens at a particular time, at a particular place, in a particular way“.^62^ Aus der englischen Rechtsprechung geht z. B. hervor, dass das Fassen eines Plans^63^ kein Ereignis im Sinne einer Aggregationsklausel darstellt. Darüber, ob eine Entscheidung, etwas zu tun, ein Ereignis sein kann, ist sich die englische Rechtsprechung nicht einig.^64^ Umstritten scheint auch, ob Unterlassungen als Ereignisse in Betracht fallen.^65^

Sofern ein Geschehnis grundsätzlich als Ereignis in Betracht fällt, ist regelmässig zu klären, ob es sich dabei um ein einziges oder mehrere separate Ereignisse handelt. In der *Dawson’s Field Arbitration* wurde diese Unsicherheit folgendermassen illustriert: „The crews of a submarine and of ships which are attacked and sunk in a convoy would no doubt regard each attack and sinking as a separate occurrence. An admiral at Naval Headquarters might regard the whole attack and its results as one occurrence; a historian almost certainly would.“^66^ Ob ein Geschehnis als ein oder zwei Ereignisse anzusehen ist, ist nach englischem Verständnis auch in Bezug auf die Rückversicherung vom Blickwinkel des Versicherungsnehmers im Erstversicherungsverhältnis zu bestimmen.^67^ In vielen Fällen dürfte die Frage, wie viele Ereignisse ein Geschehnis bilden, umstritten sein. Damit kann konstatiert werden, dass in Bezug auf das erste von Lord Justice Evans beschriebene Element eines ereignisbasierten Aggregationsmechanismus („*something that can properly be described as an event*“) Rechts*un*sicherheit herrscht.

Die Verwendung des Ereignisbegriffs als *unifying factor* indiziert gewisse Kausalitätserfordernisse einer Schadenaggregation. Das relevante Ereignis muss für alle zu aggregierenden Einzelschäden kausal sein und darf in der Kausalkette von den Einzelschäden aus betrachtet nicht allzu weit zurückliegen.^68^ Die Kausalitätsanforderungen eines Aggregationsmechanismus werden allerdings nicht alleine durch den *unifying factor*, sondern zusätzlich durch den verwendeten *linking phrase* geprägt. Auch der in ereignisbasierten Aggregationsklauseln regelmässig verwendete *linking phrase* „*arising out of*“ fordert einen eher starken Kausalzusammenhang zwischen dem relevanten Ereignis und den jeweiligen Einzelschäden.^69^ Anzumerken ist, dass die Kausalitätserfordernisse durch *unifying factor* und *linking phrase* gemeinsam bestimmt werden und dass sich die beiden Klauselbestandteile mit Blick auf die Kausalitätserfordernisse eines Aggregationsmechanismus gegenseitig beeinflussen.^70^

Verbindet eine Aggregationsklausel den *unifying factor* „*event*“ und den *linking phrase* „*arising out of*“ kann das massgebliche Ereignis weiter zurückliegen als der „*proximate cause*“ der Einzelschäden, d. h. die Verwirklichung der rückversicherten Gefahr.^71^ Entsprechend muss das für die Schadenaggregation relevante Ereignis mit dem eigentlichen Schadenereignis, d. h. mit der Verwirklichung der versicherten Gefahr, nicht identisch sein. Trotzdem muss der Kausalzusammenhang zwischen dem Ereignis und den Einzelschäden aber relativ stark sein.^72^ Nach Lord Justice Rix erfordere eine ereignisbasierte Aggregationsklausel *per definitionem* einen starken Kausalzusammenhang.^73^

Letztlich besteht allerdings kein abstrahierbarer Massstab dafür, ob der Kausalzusammenhang zwischen einem Ereignis und einer Vielzahl von Einzelschäden für eine Aggregation stark genug ist. Der englische Court of Appeal hat daher festgestellt, dass das relevante Ereignis, dasjenige sei „which should be regarded as the cause of the [individual losses to be aggregated] so as to make it appropriate to regard these as constituting for the purposes of aggregation under this policy one loss“.^74^ Mit anderen Worten soll geprüft werden, ob ein Ereignis für die Aggregation der Einzelschäden kausal genug ist, indem gefragt wird, ob eine Aggregation angebracht erscheint.

Diese Vorgehensweise erscheint unbefriedigend. Wenn man davon ausgeht, dass Einzelschäden nur aggregiert werden dürfen, wenn sie auf ein Ereignis zurückgehen, das kausal genug ist, so kann das erforderliche Mass an Kausalität nicht mit der Frage ermittelt werden, ob eine Aggregation im Einzelfall als angebracht erscheint. Denn andernfalls handelt es sich bei der Beurteilung, ob die vereinbarten Kausalitätserfordernisse im Einzelfall erfüllt sind, um reines Ermessen des Richters bzw. des Schiedsrichters.^75^

Letztlich kommt Lord Justice Rix in *Scott v Copenhagen Reinsurance Co (UK) Ltd* auch auf dieses Ergebnis. In seiner Urteilsverkündigung erklärte er:

Are the losses to be aggregated all arising from one event? That question can only be answered by finding and considering all the relevant facts carefully, and then conducting an exercise of judgment. That exercise can be assisted by considering those facts not only globally and intuitively and by reference to the purpose of the clause, but also more analytically, or rather by reference to the various constituent elements of what makes up one single unifying event. It remains an *exercise of judgment*, not a reformulation of the clause to be construed and applied.^76^

Das zweite Element eines ereignisbasierten Aggregationsmechanismus nach Lord Justice Evans, nämlich, dass zwischen dem relevanten Ereignis und den Einzelschäden Kausalzusammenhänge bestehen müssen, erscheint unproblematisch. Wie die vorangehenden Ausführungen aber zeigen, ist die für eine Schadenaggregation erforderliche Stärke der Kausalzusammenhänge und damit das dritte Element nach Lord Justice Evans unsicher.

### Der „*unities test*“

Gerichte ziehen den sog. „*unities test*“ heran, um diese schwierigen Ermessensfragen nicht nur intuitiv, sondern – wie Lord Justice Rix sagt – auch analytisch anzugehen.^77^ Der *unities test* wurde vom Schiedsgericht in der *Dawson’s Field Arbitration* entwickelt, um die Frage zu klären, ob die Umstände einer Mehrzahl von Einzelschäden jenen Grad an Verbundenheit aufweisen, der eine Aggregation rechtfertigt. Konkret hat das Schiedsgericht den Kausalverlauf (*unity of cause*), das örtliche (*unity of location*) und zeitliche (*unity of time*) Auftreten der Schadenumstände sowie die Absichten (*unity of intent*) allfälliger Schädiger untersucht.^78^

Bei genauerem Hinsehen, ergeben sich im Zusammenhang mit dem *unities test* allerdings zahlreiche Unklarheiten; insbesondere die Folgenden: grundsätzlich werden Einzelschäden aggregiert, wenn zwischen ihnen und einem Ereignis ein Kausalzusammenhang besteht.^79^ Allerdings kommt nicht jedes für die Einzelschäden kausale Ereignis als Aggregationsereignis in Betracht. Vielmehr bedarf es zwischen dem Aggregationsereignis und jedem Einzelschaden Kausalzusammenhänge einer gewissen Stärke.^80^ Das Konzept der „*unity of cause*“ greift dieses Kausalitätserfordernis auf. In *Kuwait Airways Corp v Kuwait Insurance Co SAK* scheint der englische Commercial Court befunden zu haben, dass mit der Frage, ob *unity of cause* vorliegt, lediglich geprüft wird, ob zwischen einem Ereignis und den Einzelschäden überhaupt Kausalzusammenhänge bestehen.^81^ Demgegenüber untersuchte das Schiedsgericht in *Aioi Nissay Dowa Insurance Co Ltd v Heraldglen Ltd* mittels der Rechtsfigur der *unity of cause*, ob die Kausalzusammenhänge zwischen einem Ereignis und den Einzelschäden die erforderliche Stärke aufwiesen.^82^ Daraus ergibt sich eine Unsicherheit darüber, ob *unity of cause* bereits vorliegt, wenn zwischen einem Ereignis und den Einzelschäden überhaupt Kausalzusammenhänge bestehen oder ob nur dann *unity of cause* vorliegt, wenn diese Kausalzusammenhänge eine gewisse Stärke aufweisen.

Unter dem Gesichtspunkt der „*unity of location*“ wird untersucht, ob sich die Schadensumstände durch eine gewisse örtliche Nähe auszeichnen.^83^ Tun sie dies, so spricht dies dafür, dass eine Aggregation der Einzelschäden gerechtfertigt ist.^84^ Allerdings erscheint die Rechtsprechung zur *unity of location* zu einem gewissen Grade willkürlich: in *Mann v Lexington Insurance Co* befand der englische Court of Appeal, dass die Beschädigungen von 22 Filialen einer Supermarktkette auf der indonesischen Insel Java im Rahmen von bürgerlichen Unruhen keine *unity of location* aufgewiesen haben, weil sich die Filialen an verschiedenen Orten auf der Insel befunden hatten.^85^ In *Midland Mainline Ltd v Eagle Star Insurance Co Ltd* befand der englische Commercial Court, dass nach einem Zugunglück notfallbedingte Geschwindigkeitsbegrenzungen an verschiedenen Stellen auf dem englischen Schienennetz keine *unity of location* aufgewiesen haben, weil die Begrenzungen für geografisch unterschiedliche Stellen im Netz ausgesprochen worden waren.^86^ In *Aioi Nissay Dowa Insurance Co Ltd v Heraldglen Ltd* befand ein Schiedsgericht sogar, dass die Zerstörung der Twin Towers am 11. September 2001 keine *untiy of location* aufgewiesen hatte, obwohl die beiden Türme des World Trade Centers unterirdisch miteinander verbunden gewesen waren.^87^ Demgegenüber befand das Schiedsgericht der *Dawson’s Field Arbitration*, dass die Zerstörung dreier Flugzeuge auf einem Rollfeld im jordanischen Dawson’s Field *unity of location* aufgewiesen hatte.^88^ Bemerkenswert erscheint denn auch die gerichtliche Bestätigung des Schiedsspruches in *IRB Brasil Resseguros SA v CX Reinsurance Co Ltd*, wonach die Installation von Isoliermaterial in verschiedenen Gebäuden in ganz England *unity of location* aufgewiesen hatte.^89^ Daraus ist ersichtlich, dass die Frage, ob im Einzelfall *unity of location* vorliegt, völlig im Belieben des Entscheidungsträgers steht.

Weisen die Schadenumstände „*unity of time*“ auf, so spricht dies dafür dass eine Aggregation der Einzelschäden angebracht ist.^90^ Das Konzept der *unity of time* vermag jedoch ebenfalls nicht, Rechtssicherheit zu schaffen. Denn es hat sich als extrem flexibel erwiesen. Eine Auswertung der Rechtsprechung dazu ergibt, dass Entscheidungsträger praktisch in jedem Fall Gründe für und gegen das Vorliegen von *unity of time* nennen können.^91^ In der *Dawson’s Field Arbitration* befand das Schiedsgericht, die Zerstörung dreier entführter Flugzeuge weise *unity of time* auf, weil die entsprechenden Explosionen in einer Zeitspanne von wenigen Minuten erfolgt waren.^92^ Offenbar unerheblich war die Frage, zu welchem Zeitpunkt die Flugzeuge entführt und nach Dawson’s Field geflogen worden waren. Ein anderes Schiedsgericht vertrat demgegenüber die Meinung, die Anschläge auf den Nordturm bzw. den Südturm des World Trade Centers am 11. September 2001 wiesen keine *unity of time* auf. Zu diesem Schluss kam es, weil es seiner Beurteilung den gesamten Zeitraum zwischen dem Check-in der Attentäter vor der Entführung der Flugzeuge und den Einschlägen der Maschinen in die Türme zugrunde gelegt hatte.^93^ Dieser Vergleich zeigt, dass die Frage, ob *unity of time* vorliegt, masseglich vom der Beurteilung zugrunde gelegten Zeitraum abhängt. Solange die Entscheidungsträger diesen Zeitraum beliebig bestimmen können, schafft das Kriterium der *unity of time* keine Rechtssicherheit.^94^

Es erstaunt nicht, dass auch die Rechtsfigur der „*unity of intent*“ ungeeignet ist, im Bereich der ereignisbasierten Schadenaggregation Rechts*un*sicherheit auszuräumen. Nach der Vorstellung der Entwickler des *unities test* spricht eine gemeinsame Absicht der Verursacher der Einzelschäden dafür, letztere zu aggregieren.^95^ Allerdings geht aus der englischen Rechtsprechung nicht hervor, was die Verursacher genau beabsichtigen müssen, damit eine Aggregation der Einzelschäden in Frage kommt. Konkret stellt sich die Frage, ob diese Absichten auf die Verursachung der verschiedenen Einzelschäden gerichtet sein müssen oder ob es ausreicht, wenn sie auf eine Handlung gerichtet sind, aus der in der Folge – möglicherweise auch völlig unbeabsichtigt – eine Mehrzahl von Einzelschäden hervorgeht.^96^ In *Kuwait Airways Corp v Kuwait Insurance Co SAK* befand das Gericht, bei der Invasion von Kuwait durch den Irak und der Aneignung von 15 Flugzeugen durch irakische Militärs habe *unity of intent* vorgelegen. Denn es sei Saddam Husseins Absicht gewesen, Kuwait Airways die Verfügungsgewalt über die Flugzeuge zu entziehen und sich den Wert derselben anzueignen. Seine Absicht war damit auf die Verursachung einer Mehrzahl von Einzelschäden gerichtet.^97^ In der *Dawson’s Field Arbitration* entschied das Schiedsgericht, es habe *unity of intent* vorgelegen, weil die Absichten der Flugzeugentführer auf die Zerstörung aller drei Flugzeuge in Dawson’s Field gerichtet war.^98^ In *Midland Mainline Ltd v Eagle Star Insurance Co Ltd* entschied der englische Commercial Court, die Tatsache, dass aufgrund eines Unfalles an verschiedenen Stellen eines Eisenbahnnetzwerkes Geschwindigkeitsbegrenzungen angeordnet worden waren, würde eine *unity of intent* begründen.^99^ Unzweifelhaft wollte man mit der Anordnung dieser Geschwindigkeitsbeschränkungen weitere Unfälle vorbeugen und nicht, den Eisenbahngesellschaften Schäden verursachen.^100^ Damit ist nicht ersichtlich, wie die Absichten der Schadenverursacher gelagert sein müssen, damit man von einer *unity of intent* ausgehen kann.

Zudem haben nicht alle (Schieds‑)Gerichte, die eine Frage der ereignisbasierten Aggregation zu beurteilen hatten, den *unities test* überhaupt angewandt.^101^ In *MIC Simmonds v Gammell* erklärte der englische Commercial Court sogar ausdrücklich, „[t]he ‚unities‘ are merely an aid in determining whether the circumstances of the losses involved such a degree of unity as to justify their being described as ‚arising out of one occurrence‘“.^102^ Ergibt sich aus anderen Gründen, dass zwischen dem Ereignis und den Einzelschäden genügend starke Kausalzusammenhänge bestehen, muss der *unities test* offenbar nicht durchgeführt werden.^103^ In welchen Fällen eine analytische Herangehensweise an die Ausübung des Ermessen durch die Anwendung des *unities test* zu erfolgen hat, bleibt damit aber unklar.

Der *unities test* scheint nicht geeignet zu sein, die Rechts*un*sicherheit in der ereignisbasierten Schadenaggregation zu verringern bzw. die Ermessensausübung der Entscheidungsträger zu objektivieren. Denn letztlich – so bestätigte das Schiedsgericht in *Aioi Nissay Dowa Insurance Co Ltd v Heraldglen Ltd* – ist auch die Durchführung des *unities test* eine Ermessensangelegenheit.^104^

### Fazit zur ereignisbasierten Schadenaggregation nach englischem Recht

Nach Lord Justice Evans besteht ein ereignisbasierter Aggregationsmechanismus aus drei Elementen. Die Beurteilung, ob etwas passiert ist, das man als Ereignis bezeichnen kann (erstes Element) birgt bereits erhebliche Rechts*un*sicherheit. Denn darüber, welche Geschehnisse als Ereignisse im Sinne einer Aggregationsklausel anzusehen sind, herrscht keine Einigkeit. Ebenfalls unklar ist, ob ein Geschehnis im Einzelfall aus einer Aneinanderreihung mehrerer Ereignisse besteht oder selber ein einziges Ereignis darstellt.

Auch Lord Justice Evans drittes Element, nämlich dass die Kausalzusammenhänge zwischen einem Ereignis und den Einzelschäden eine gewisse Stärke^105^ aufweisen müssen, stellt (Schieds‑)Gerichte immer wieder vor Probleme. Letzten Endes handelt es sich dabei um eine Ermessensfrage. Zur Strukturierung und einer gewissen Objektivierung ihrer Ermessensausübung wenden (Schieds‑)Gerichte regelmässig den *unities test* an. Eine Analyse der Rechtsprechung zum *unities test* zeigt jedoch, dass dieser selber zur Ermessensfrage verkommt und nicht geeignet ist, im Bereich der ereignisbasierten Schadenaggregation Rechtssicherheit zu schaffen.

Unter englischem Recht besteht im Bereich der ereignisbasierten Schadenaggregation damit erhebliche Rechts*un*sicherheit; eine Rechtsunsicherheit, die der eingangs erwähnten, erheblichen Bedeutung der nichtproportionalen Rückversicherung nicht gerecht wird.

## Ursachenbasierte Schadenaggregation (*cause-based aggregation*)

Ursachenbasierte Aggregationsklauseln finden sich häufiger in Erstversicherungs- als in Rückversicherungsverträgen.^106^ Gelegentlich wird die Aggregationsklausel eines Erstversicherungsvertrags jedoch durch Verweis in den Rückversicherungsvertrag inkorporiert. Deshalb ist es durchaus auch möglich, in Rückversicherungsverträgen ursachenbasierte Aggregationsklauseln vorzufinden.^107^

Ursachenbasierte Aggregationsklauseln enthalten typischerweise entweder das Verbindungsmerkmal (*unifying factor*) des „*originating cause or source*“^108^ oder jenes des „*one source or original cause*“^109^. Die beiden Varianten werden von Literatur und Rechtsprechung als funktional identisch erachtet.^110^ In der Regel wird dieses Verbindungsmerkmal entweder zusammen mit dem *linking phrase* „*consequent upon or attributable to*“^111^ oder „*arising from*“^112^ verwendet.

In *Axa Reinsurance (UK) Ltd v Field* grenzte Lord Mustill im House of Lords das Verbindungsmerkmal der Ursache (*cause*) von jenem des Ereignisses (*event*) ab. Während ein Ereignis etwas sei, das zu einer bestimmten Zeit an einem bestimmten Ort in einer bestimmten Art und Weise geschehe, sei der Ursachenbegriff viel weitläufiger. Beispielsweise könne ein Zustand und sogar die Tatsache, dass nichts geschehe, als Ursachen im Sinne einer Aggregationsklausel verstanden werden.^113^ Dem hielt Justice Morison in *Countrywide Assured Group Plc v Marshall* entgegen, der Ursachenbegriff sei nicht einfach nur weitläufiger als der Begriff des Ereignisses. Vielmehr beschreibe das Wort „Ereignis“ etwas, das passiere, während das Wort „Ursache“ erkläre, warum etwas geschehe.^114^

Das bedeutet jedoch nicht, dass Ursache und Ereignis unter keinen Umständen zusammenfallen können.^115^ Es ist davon auszugehen, dass jedes für die Schadenaggregation relevante Ereignis gleichzeitig als Ursache bezeichnet werden kann.^116^ So sind z. B. die Erdbeben von Christchurch im Jahre 2011 sowohl als Ursache(n) im Sinne einer ursachenbasierten Aggregationsklausel als auch als Ereignis(se) im Sinne einer ereignisbasierten Aggregationsklausel zu verstehen.^117^ Umgekehrt kann nicht jede Ursache auch als Ereignis qualifiziert werden. So wäre es beispielsweise undenkbar, die Anfälligkeit Neuseelands auf Erdbeben als Ereignis zu verstehen.^118^ Als Ursache im Sinne einer Aggregationsklausel fällt diese Anfälligkeit nach Merkin demgegenüber in Betracht.^119^

In *Axa Reinsurance (UK) Ltd v Field* vertrat Lord Mustill die Ansicht, „the word ‚originating‘ was […] consciously chosen to open up the widest possible search for a unifying factor in the history of the losses which it is sought to aggregate.“^120^ Allerdings – zu diesem Schluss gelangte jedenfalls der Commercial Court in *American Centennial Insurance Co v Insco Ltd* – erfordert auch die ursachenbasierte Schadenaggregation, das zwischen der Ursache und den Einzelschäden Kausalzusammenhänge einer gewissen Stärke bestehen.^121^

Unbestritten erscheint, dass die Kausalitätserfordernisse in ursachenbasierten Aggregationsmechanismen im Vergleich mit jenen in ereignisbasierten weniger streng sind. Mit anderen Worten erlaubt ein ursachenbasierter Aggregationsmechanismus eine Schadenaggregation noch, wenn die Kausalzusammenhänge zwischen dem Verbindungsmerkmal und den Einzelschäden für einen ereignisbasierten Aggregationsmechanismus bereits zu schwach wären.^122^

In einem Fall von haftungsbewehrten Beratungsfehlern wurde z. B. die mangelnde Ausbildung des Beratungsteams als für die Schadenaggregation relevante Ursache angesehen.^123^ Unter gegebenen Umständen kann ein Missverständnis zwischen Personen als Ursache für die Entstehung einer Vielzahl von Einzelschäden gelten.^124^ Weiter wurde das Fehlen eines adäquaten Sicherheitskonzepts der Betreiberin eines Hafens als Ursache für zahlreiche Schäden an auf der Hafenanlage gelagertem Material als Ursache im Sinne einer Aggregationsklausel angesehen.^125^ Wie bereits erwähnt, sieht Merkin sogar die Tatsache, dass Neuseeland in einem Erdbebengebiet liegt, als mögliche Ursache für eine Vielzahl von Erdbebenschäden an.^126^

Es erscheint zwar denkbar, dass aus einem Zustand mehrere Einzelschäden resultieren.^127^ So könnte man sagen, dass der Zustand der Unkenntnis eines Versicherungs-Underwriters über die Risiken von Asbest für eine Deckungspflicht des Versicherers für unzählige Asbestschäden ursächlich war.^128^ Demgegenüber erscheint es eher zweifelhaft, ob die Anfälligkeit einer Insel auf Erdbebenschäden als deren Ursache angesehen werden kann.^202^ In diesem Sinne dürfte in vielen Situation umstritten sein, ob etwas als Ursache im Sinne einer Aggregationsklausel qualifiziert.

Aber selbst wenn eine Ursache für eine Vielzahl von Schäden ausgemacht werden kann, besteht noch immer die Unsicherheit, ob die Stärke der Kausalzusammenhänge zwischen dieser Ursache und den Einzelschäden für eine Schadenaggregation ausreicht. Im Bereich der ursachenbasierten Schadenaggregation ist der *unities test* jedenfalls nicht anwendbar.^129^ Es scheint, als ob englische Gerichte für diese Fragen bisher keine Beurteilungskriterien entwickelt hätten.

Damit besteht auch mit Blick auf die ursachenbasierte Schadenaggregation erhebliche Rechts*un*sicherheit.

## Schadenaggregation unter den PRICL 2019

### Wissenschaftliches Kodifikationsprojekt

Rechtsunsicherheit besteht nicht nur im Bereich der Schadenaggregation, sondern auch in vielen anderen Themengebieten des Rückversicherungsvertragsrechts.^130^ Unter der Leitung von Prof. Helmut Heiss^131^, Prof. Martin Schauer^132^ und Prof. Manfred Wandt^133^ arbeiten Wissenschaftler aus aller Welt^134^, Marktvertreter von Erst- und Rückversicherern sowie Rückversicherungsmakler^135^ seit 2016 an einem wissenschaftlichen Kodifikationsprojekt, in dem für viele Themenbereiche des Rückversicherungsvertragsrechts möglichst rechtssichere Regeln erarbeitet werden.^136^ Die sog. „PRICL-Gruppe“ führt das Projekt in Kooperation mit dem im Bereich der Rechtsvereinheitlichung renommierten International Institute for the Unification of Private Law (UNIDROIT).^137^ Die erste Projektphase wurde vom Schweizerischen Nationalfonds (SNF), von der Deutschen Forschungsgemeinschaft (DFG) und vom Österreichischen Fonds zur Förderung der Wissenschaftlichen Forschung (FWF) finanziell unterstützt und mündete Ende 2019 in die Publikation der ersten fünf Kapitel der Principles of Reinsurance Contract Law (PRICL), den sog. „PRICL 2019“.^138^ In einer zweiten Projektphase – weiterhin unterstützt vom SNF und der DFG – arbeitet die PRICL-Gruppe bis Ende Juli 2022 an Prinzipien zu weiteren Fragen des Rückversicherungsvertragsrechts.

Im ersten Kapitel der PRICL 2019 findet sich ein allgemeiner Teil, indem u. a. die Anwendbarkeit und die Auslegung der Prinzipien und ihr Verhältnis zu anderen Rechtsquellen geregelt wird. Kapitel 2 widmet sich den Pflichten des Zedenten und des Rückversicherers. Für den Fall, dass die Vertragsparteien gegen ihre Pflichten verstossen, hält Kapitel 3 ausgewogene Rechtsbehelfe bereit. Sodann enthält Kapitel 4 Regeln zur Allokation einzelner Schäden in die richtige Versicherungsperiode.

Regeln zur ereignis- und ursachenbasierten Schadenaggregation werden in Kapitel 5 statuiert. Der Anspruch der PRICL, im Bereich der Schadenaggregation vorhandene Rechtsunsicherheit wenigstens teilweise zu beseitigen, wird im offiziellen Kommentar zu Art. 5.1 PRICL zum Ausdruck gebracht.^139^ Im Folgenden sollen die für die PRICL entwickelten Konzepte der ereignis- und ursachenbasierten Schadenaggregation überblicksartig vorgestellt werden.

### Ereignisbasierte Schadenaggregation unter Art. 5.2 PRICL

Art. 5.2 PRICL ist anwendbar, sofern sich die Parteien für ihren Rückversicherungsvertrag auf einen ereignisbasierten Aggregationsmechanismus geeinigt haben.^140^ Die Bestimmung lautet wie folgt:

(1) Where the parties agree on an event-based aggregation in a contract reinsuring first-party insurance policies, all losses that occur as a direct consequence of the same materialization of a peril reinsured against shall be considered as arising out of one event.

(2) Where the parties agree on an event-based aggregation in a contract reinsuring third-party liability insurance policies, all losses that occur as a direct consequence of the same act, omission or fact giving rise or allegedly giving rise to the primary insured’s liability shall be considered as arising out of one event.

Aus der Bestimmung geht ohne weiteres hervor, dass eine Schadenaggregation nur dann in Betracht fällt, wenn ein Ereignis für zwei oder mehr Einzelschäden kausal ist.^141^ Das Konzept der Bestimmung basiert auf der Erkenntnis, dass es vielfach umstritten ist, ob ein Geschehnis als Ereignis im Sinne einer Aggregationsklausel zu qualifizieren ist und dass es praktisch unmöglich sein dürfte, in einer abstrakten Regel festzulegen, wie weit ein Ereignis auf der Kausalkette zurückliegen darf, um für die Schadenaggregation noch relevant zu sein. Mit anderen Worten versucht Art. 5.2 PRICL sowohl das erste als auch das dritte von Lord Justice Evans^142^ angeführte Beurteilungskriterium zu vereinfachen.^143^ Der unter englischem Recht entwickelte, rechtsunsichere *unities test* muss unter Anwendung von Art. 5.2 PRICL nicht bemüht werden.^144^

#### Bestimmung des für die Schadenaggregation relevanten Ereignisses

Für die Bestimmung des für die Schadenaggregation relevanten Ereignisses unterscheiden die PRICL zwischen der Rückversicherung von Sachversicherungspolicen^145^ (Abs. 1) und jener von Haftpflichtpolicen (Abs. 2).

##### 7.2.1.1 Art. 5.2 (1) PRICL – Rückversicherung von Sachversicherungspolicen

Die Sachversicherung versichert in der Regel das Risiko, dass der Versicherte durch die Verwirklichung einer im Vertrag designierten Gefahr in seinem Eigentum geschädigt wird.^146^ Der Erstversicherer wird gegenüber seinem Versicherungsnehmer leistungspflichtig, wenn sich eine solche Gefahr verwirklicht und dadurch ein Schaden eintritt.^147^ Soweit sich dadurch auch eine im Rückversicherungsvertrag definierte Gefahr verwirklicht, wird der Rückversicherer gegenüber seinem Zedenten (Erstversicherer) leistungspflichtig.

Lord Justice Evans erstes Element eines ereignisbasierten Aggregationsmechanismus fragt danach, ob ein Geschehnis als Ereignis bezeichnet werden kann („*can properly be described as an event*“). Art. 5.2 (1) PRICL löst die diesbezügliche Rechtsunsicherheit auf, indem es nur die Verwirklichung einer rückversicherten Gefahr als Ereignis anerkennt („*materialization of a peril reinsured against*“).^148^ Ein jedes Geschehnis, das nicht als Verwirklichung einer rückversicherten Gefahr bezeichnet werden kann, begründet kein Ereignis für die Schadenaggregation i. S. v. Art. 5.2 (1) PRICL.

Weil es möglich ist, dass sich die rückversicherte Gefahr in kurzer Zeit mehrmals verwirklicht, ist es wichtig, dass jede Verwirklichung separat betrachtet wird. Laut Art. 5.2 (1) PRICL sind nur jene Einzelschäden zu aggregieren, die sich aus derselben Verwirklichung einer rückversicherten Gefahr ergeben haben („*same materialization*“). Die Herausforderung besteht also in der Ermittlung einer einzelnen Gefahrverwirklichung. Gerade bei Naturgefahren wie zum Beispiel einem Erdbeben mit potentiellen Nachbeben kann es durchaus umstritten sein, ob sich die Gefahr ein einziges Mal oder wiederholt verwirklicht hat. Solche Fragen werden i. d. R. naturwissenschaftlich begutachtet. Auch der offizielle Kommentar zu den PRICL spricht sich für eine wissenschaftliche Untersuchung dieser Fragen im Einzelfall aus.^149^

Sofern das rückversicherte Risiko nicht in einer Naturgefahr, sondern vielmehr in der Gefahr eines durch Menschenhand verursachten Schadens liegt, dürfte es regelmässig schwierig sein, anhand von wissenschaftlichen Kriterien zu entscheiden, ob sich eine Gefahr einmal oder mehrmals verwirklicht hat.^150^ Wissenschaftliche Aussagen darüber, wie viele Ereignisse in den Terroranschlägen vom 11. September 2001 auf das World Trade Center zu sehen sind, lassen sich nicht treffen. Laut dem offiziellen Kommentar zu den PRICL soll die Frage aus der Perspektive vernünftiger Parteien im Zeitpunkt, in welchem der Rückversicherungsschutz ausgehandelt worden ist, beantwortet werden.^151^

##### 7.2.1.2 Art. 5.2 (2) PRICL – Rückversicherung von Haftpflichtversicherungspolicen

Haftpflichtpolicen bieten Schutz gegen die Haftbarkeit des Erstversicherungsnehmers gegenüber Dritten.^152^ Sie nennen oftmals keine versicherten Gefahren.^153^ Dasselbe gilt für die Rückversicherungsvereinbarung, die für Haftpflichtrisiken Rückdeckung bietet. Aus diesem Grund ist nach Art. 5.2 (2) PRICL für die Bestimmung eines Ereignisses nicht die Verwirklichung einer im Rückversicherungsvertrag designierten Gefahr massgeblich.

Vielmehr liegt ein Ereignis in diesem Fall in der Haftpflicht auslösenden Verfehlung des Erstversicherungsnehmers („*act, omission or fact giving rise […] to the primary insured’s liability*“). Die Formulierung stellt zunächst klar, dass ein Ereignis in der Haftpflicht auslösenden Tatsache zu suchen ist und Geschehnisse, die auf der Kausalkette weiter zurückliegen, irrelevant sind.^154^ Sodann anerkennt die Regelung in Art. 5.2 (2) PRICL ausdrücklich, dass ein Ereignis nicht nur in einem aktiven Tun des Erstversicherungsnehmers, sondern unter gegebenen Umständen auch in dessen Unterlassung liegen kann. Eine Unterlassung wird dann zum Ereignis i. S. v. Art. 5.2 (2) PRICL, wenn in ihr eine Haftpflicht begründende Pflichtverletzung liegt.^155^ Sofern die Unterlassung ohne weiteres aktives Zutun des Erstversicherungsnehmers dagegen nicht zu einem Ersatzanspruch des geschädigten Dritten führt, begründet die Unterlassung kein Ereignis.^156^ Die Formulierung von Art. 5.2 (2) PRICL räumt damit die im englischen Recht bestehende Unsicherheit aus, ob Unterlassungen Ereignisse i. S. einer Aggregationsklausel darstellen können.

Wird ein Erstversicherungsnehmer unter dem Titel einer Gefährdungshaftung ersatzpflichtig, so begründet jene Tatsache, welche die Haftpflicht auslöst, ein Ereignis i. S. v. Art. 5.2 (2) PRICL.^157^ Damit ist ein Ereignis i. S. v. Art. 5.2 (2) PRICL auch denkbar, wenn der Erstversicherungsnehmer weder eine schädigende Handlung noch eine schädigende Unterlassung begangen hat.

#### Das Kriterium der „direct consequence“

Beide Absätze von Art. 5.2 PRICL lassen eine Schadenaggregation nur zu, sofern die betreffenden Einzelschäden die direkte Folge („*direct consequence*“) eines Ereignisses darstellen. Wie bereits erwähnt, bedarf es für eine ereignisbasierte Schadenaggregation auch unter den PRICL Kausalzusammenhänge zwischen einem Ereignis und den Einzelschäden.^158^

Die Formulierung der Bestimmung erweckt den Eindruck, dass nur qualifizierte, nämlich direkte Kausalzusammenhänge eine Schadenaggregation ermöglichen. Sofort wird man fragen müssen, was als direkter bzw. indirekter Einzelschaden gilt und zu bedenken geben, dass die erforderliche Stärke der Kausalzusammenhänge auch unter Art. 5.2 PRICL rechtsunsicher erscheine.

Dem ist allerdings entgegenzuhalten, dass für die Beurteilung, ob ein Einzelschaden die direkte Folge eines Ereignisses ist, kein allzu abstrakter, undefinierbarer Massstab zur Anwendung gelangen soll. Nach dem offiziellen Kommentar zu Art. 5.2 PRICL ist ein Einzelschaden als direkte Folge eines Ereignisses anzusehen, sofern er eine unausweichliche Folge des Ereignisses darstellt und kein entscheidender, unabhängiger Faktor hinzutritt, ohne den der Einzelschaden nicht eingetreten wäre.^159^ Ein solcher massgeblicher, unabhängiger Faktor, der zum Ereignis hinzutritt und ohne den der Einzelschaden nicht eingetreten wäre, kann darin bestehen, dass eine Person es unterlässt, das Ereignis abzuwenden, obwohl dazu eine Pflicht bestanden hätte (*conditio cum qua non*).^160^ Ebenso gut kann ein Geschehnis oder die aktive Handlung einer Person einen solchen Faktor darstellen (*conditio sine qua non*).^161^

Selbst wenn ein Einzelschaden neben dem Ereignis von einem unabhängigen Einzelschaden mitgeprägt worden ist, entfällt eine Schadenaggregation nur, sofern dieser Faktor für den Schadeneintritt von entscheidender Bedeutung war.^162^ Beispielsweise verhindert die Tatsache, dass ein Feuer nur aufgrund windiger Verhältnisse auf ein bestimmtes Grundstück übergetreten ist, eine Schadenaggregation nicht, sofern diese Windverhältnisse für den fraglichen Ort nicht gänzlich ungewöhnlich sind.^163^

Eine Mehrheit von Einzelschäden ist auch zu aggregieren, sofern das fragliche Ereignis in der Kausalkette nicht unmittelbar vor dem Eintritt der Einzelschäden stattfindet. Es ist durchaus denkbar, dass das Ereignis unweigerlich zu einem weiteren Geschehnis führt, welches dann seinerseits unvermeidbar im Eintritt der Schäden mündet.^164^ Unter Art. 5.2 PRICL gilt der Einzelschaden in einem solchen Fall als direkte Folge des Ereignisses.

Damit dürften die Kausalitätserfordernisse unter Art. 5.2 PRICL mit weit weniger Rechtsunsicherheit verbunden sein als jene unter englischem Recht. Ob sich Lord Justice Evans drittes Element einer ereignisbasierten Schadenaggregation durch Art. 5.2 PRICL in der Praxis tatsächlich optimieren lässt, wird sich zeigen müssen.

### Ursachenbasierte Schadenaggregation unter Art. 5.3 PRICL

Haben die Vertragsparteien einen ursachenbasierten Aggregationsmechanismus vereinbart, so gelangt Art. 5.3 PRICL zur Anwendung. Die Bestimmung lautet wie folgt:

(1) Where the parties agree on a cause-based aggregation in a contract reinsuring first-party insurance policies, all losses that occur as the direct consequence of one or multiple events within the meaning of Article 5.2 paragraph (1) shall be considered as arising out of one common cause if it was reasonably foreseeable that a cause of this kind could give rise to such an event.

(2) Where the parties agree on a cause-based aggregation in a contract reinsuring third-party liability insurance policies, all losses that occur as the direct consequence of one or multiple events within the meaning of Article 5.2 paragraph (2) shall be considered as arising out of one common cause if it was reasonably foreseeable that a cause of this kind could give rise to such an event.

Wie Art. 5.2 PRICL unterscheidet auch Art. 5.3 PRICL zwischen der Rückversicherung von Sachversicherungspolicen (Abs. 1) und jener von Haftpflichtpolicen (Abs. 2).^165^ Art. 5.3 PRICL folgt der englischen Rechtsposition auch darin, dass eine Ursache für eine Schadenaggregation in der Kausalkette weiter zurückliegen darf als ein Ereignis.^166^ Die PRICL versuchen, die Kausalitätserfordernisse einer ursachenbasierten Schadenaggregation in einer abstrakten Regel zu beschreiben.

#### Mehrere Verwirklichungen rückversicherter Gefahren

Eine Ursache ist unter Umständen geeignet, die Verwirklichung einer Mehrzahl rückversicherter Gefahren zu provozieren. Beispielsweise ist es denkbar, dass ein Erdbeben sowohl einen Tsunami als auch den Ausbruch eines Feuers nach sich zieht. Unter Vorbehalt des Vorliegens weiterer Voraussetzungen gebietet Art. 5.3 PRICL, dass alle Einzelschäden – ungeachtet dessen, ob sie durch das Erdbeben selbst, den Tsunami oder das Feuer verursacht worden sind – zu aggregieren.^167^

Um sicherzustellen, dass Entscheidungsträger unter einem ursachenbasierten Aggregationsmechanismus eine entsprechend weite Schadenaggregation zulassen, sieht Art. 5.3 PRICL vor, dass zunächst alle Ereignisse i. S. v. Art. 5.2 PRICL und damit alle Gefahrverwirklichungen (Abs. 1) bzw. Haftpflicht begründenden Handlungen, Unterlassungen oder Tatsachen (Abs. 2) ermittelt werden.^168^ Auf der Suche nach einer gemeinsamen Ursache kann von den einzelnen Ereignissen aus auf den Kausalketten weiter zurückgeschritten werden.^169^ Sofern die verschiedenen Ereignisse keine gemeinsame Ursache haben, können die aus den verschiedenen Ereignissen resultierenden Einzelschäden nicht aggregiert werden. In diesem Fall gelten die einzelnen Ereignisse als Ursachen^170^, sodass all jene Einzelschäden aggregiert werden, die aus derselben Gefahrverwirklichung bzw. aus derselben Haftpflicht begründenden Handlung, Unterlassung oder Tatsache resultiert sind.^171^

Wenn die verschiedenen Ereignisse demgegenüber eine gemeinsame Ursache aufweisen, so kommt diese Ursache als Verbindungsmerkmal gemäss Art. 5.3 PRICL grundsätzlich in Betracht.^172^ Liegt diese gemeinsame Ursache in der Kausalkette zu weit zurück, so erscheint es allerdings nicht angezeigt, sie als Verbindungsmerkmal für eine Schadenaggregation anzuerkennen. Denn andernfalls wäre es z. B. möglich, die Entstehung der Erde als gemeinsame Ursache eines Erdbebens in Asien und einer Flut in Nordamerika anzugeben und entsprechend Einzelschäden zu aggregieren, die bei Lichte betrachtet, gänzlich unverbunden sind.

#### Kausalitätserfordernisse unter Art. 5.3 PRICL

Art. 5.3 PRICL bestimmt die Kausalitätserfordernisse einer ursachenbasierten Schadenaggregation durch das Kriterium der Vorhersehbarkeit („*reasonable foreseeability*“).^173^ Dieses Kriterium beschränkt, wie weit zurück eine Ursache in einer Kausalkette liegen darf, damit sie als Verbindungsmerkmal für eine Schadenaggregation noch in Betracht fällt.^174^

Sofern im Zeitpunkt des Vertragsschlusses objektiv voraussehbar war, dass eine Ursache der fraglichen Art nach dem gewöhnlichen Lauf der Dinge geeignet ist, ein Ereignis der betreffenden Art zu bewirken, ist das Kriterium erfüllt.^175^ Beispielsweise ist durchaus vorhersehbar, dass ein Erdbeben einen Tsunami hervorbringen kann. Ebenfalls vorhersehbar ist, dass ein Erdbeben den Ausbruch eines Feuers auszulösen vermag. Daher kann ein Erdbeben unter Umständen als Ursache einer Vielzahl von unter Art. 5.3 PRICL zu aggregierenden Tsunami- und Feuerschäden angesehen werden.

In gewissen angelsächsischen Rechtsordnungen wird das Rechtsinstitut der Vorhersehbarkeit („*foreseeability*“) dazu verwendet, die Haftbarkeit einer pflichtverletzenden Person bzw. eines Versicherten einzuschränken. Der Vorhersehbarkeit gemäss Art. 5.3 PRICL kommt allerdings nicht dieselbe Funktion zu. Konkret geht es nicht direkt darum, ob ein bestimmter Schaden unter dem Rückversicherungsvertrag gedeckt ist, weil er vorhersehbar war.^176^ Vielmehr soll anhand der Vorhersehbarkeit die Frage beurteilt werden, ob eine Ursache in der Kausalkette zu weit hinter einer Gefahrverwirklichung bzw. einer Haftpflicht begründenden Handlung, Unterlassung oder Tatsache zurückliegt und daher als Verbindungsmerkmal ausscheidet oder ob die Kausalzusammenhänge zwischen einer Ursache und einer Mehrzahl von Ereignissen für eine Aggregation von Einzelschäden, die sich aus verschiedenen Gefahrverwirklichungen bzw. Haftpflicht begründenden Handlungen, Unterlassunen oder Tatsachen ergeben haben, stark genug sind.^177^

Die Beurteilung, ob vorhersehbar war, dass die Ursache einer bestimmten Art zur Verwirklichung einer bestimmten, rückversicherten Gefahr führt oder eine Haftpflicht begründende Handlung, Unterlassung oder Tatsache hervorruft, dürfte in vielen Fällen kontrovers sein. Letztlich werden (Schieds‑)Gerichte diesbezüglich einen Ermessensentscheid treffen müssen. Die Kommentare und Illustrationen zu Art. 5.3 PRICL wollen die Ausübung des Ermessen strukturieren und objektivieren. Die in der ursachenbasierten Schadenaggregation angelegte Rechts*un*sicherheit kann damit sicherlich nicht restlos ausgeräumt werden. Im Vergleich zur englischen Rechtslage dürfte der Ansatz von Art. 5.3 PRICL diese Rechts*un*sicherheit jedoch merklich reduzieren.

## Fazit

Wenn die Parteien eines Rückversicherungsvertrags eine Priorität oder ein Deckungslimit vereinbaren, bestimmen sie den quantitativen Deckungsbereich des Rückversicherungsschutzes. In Abhängigkeit des quantitativen Deckungsbereichs sowie weiterer Aspekte setzen sie die Rückversicherungsprämie fest. Sofern die Parteien nicht verstehen, wie sich der an Priorität und Deckungslimit zu messende Schaden zusammensetzt, kann dies das Gleichgewicht zwischen dem quantitativen Deckungsumfang und der Rückversicherungsprämie beeinträchtigen. Eine genaue Bewertung des rückversicherten Risikos setzt entsprechend ein genaues Verständnis dessen voraus, was als ein Schaden anzusehen ist.

Weder die deutsche noch die schweizerische Judikatur hat in Bezug auf die Schadenaggregation in der Rückversicherung konkrete Regeln entwickelt. Demgegenüber haben englische Gerichte seit ca. 1995 vermehrt die Gelegenheit, Fragen der Schadenaggregation zu beurteilen. Es ist davon auszugehen, dass sich auch deutsche und schweizerische Gerichte – wenn sie denn dazu Gelegenheit erhalten – von der englischen Rechtsprechung inspirieren lassen werden.

Eine Analyse der englischen Rechtsprechung zur Schadenaggregation in der Rückversicherung hat gezeigt, dass in diesem Bereich eine erhebliche Rechts*un*sicherheit besteht. Aufgrund von terminologischen und konzeptionellen Gründen scheint zunächst unklar, was überhaupt ein Einzelschaden ist. Sodann erscheinen sowohl der Ereignisbegriff als auch der Ursachenbegriff alles andere als eindeutig. Kann in einer Unterlassung wirklich unter keinen Umständen ein Ereignis gesehen werden? Können Entscheidungen, etwas zu tun, Ereignisse darstellen? Und kann die Tatsache, dass Neuseeland in einem Erdbebengebiet liegt, wirklich als Ursache für Einzelschäden angesehen werden?

Sowohl in der ereignisbasierten als auch in der ursachenbasierten Schadenaggregation liegen die grössten Unsicherheiten in den Kausalitätserfordernissen zwischen einem *unifying factor* und den Einzelschäden. Aus der englischen Rechtsprechung scheint kein abstrahierbares Konzept zur Bestimmung dieser Kausalitätserfordernisse hervorgegangen zu sein. Lord Justice Rix erklärt in *Scott v The Copenhagen Reinsurance Co (UK) Ltd*, man müsse sich im Einzelfall die Frage stellen, ob ein Ereignis vorliege, das für eine Vielzahl von Einzelschäden ursächlich gewesen sei und eine Aggregation dieser Schäden daher angemessen erscheine.^178^

Damit nicht der Anschein entsteht, dass (Schieds‑)Gerichte Fragen der Schadenaggregation völlig intuitiv beantworten, wenden sie den „analytischen“ *unities test* an. Die Untersuchung hat allerdings gezeigt, dass auch die Anwendung des *unities test *eine sehr intuitive Angelegenheit ist. Letztlich lässt sich mit dem *unities test* ohne grössere Schwierigkeiten jedes Ergebnis begründen, sodass es letztlich eben doch wieder auf das Bauchgefühl des (Schieds‑)Richters ankommt. Dieses Ergebnis zeigt, dass es das englische Recht den Parteien nicht erlaubt, den Einfluss eines Aggregationsmechanismus auf das rückversicherte Risiko auch nur annähernd rechtssicher abzuschätzen und die Rückversicherungsprämie entsprechend festzusetzen.

Kapitel 5 der PRICL sieht Regeln zur Schadenaggregation in der Rückversicherung vor. Unsicherheiten in Bezug auf die Definition von Einzelschäden werden darin allerdings nicht ausgeräumt. Demgegenüber enthält das Kapitel sowohl für die ereignisbasierte als auch für die ursachenbasierte Schadenaggregation eigens entwickelte Konzepte, welche zur Erhöhung der Rechtsicherheit beitragen sollen. Art. 5.2 PRICL sieht vor, dass das für die Schadenaggregation relevante Ereignis in der Verwirklichung einer rückversicherten Gefahr bzw. in einer die Haftpflicht des Erstversicherungsnehmers begründenden Handlung, Unterlassung oder Tatsache liegt. Damit entfällt in der Regel die schwierige Frage, ob etwas ein Ereignis ist oder nicht. Einzig, wenn der Vertrag die rückversicherte Gefahr nicht nennt, bleibt eine kleine Ungewissheit.^203^ Art. 5.2 PRICL verzichtet ebenfalls auf komplizierte Kausalitätserfordernisse. Entsprechend dürfte die ereignisbasierte Schadenaggregation in Art. 5.2 PRICL im Verhältnis zur jener unter englischem Recht merklich rechtssicherer sein.

Wie im englischen Recht lässt auch die ursachenbasierte Schadenaggregation unter Art. 5.3 PRICL im Verhältnis zu einer ereignisbasierten Schadenaggregation eine weitreichendere Schadenaggregation zu. Konkret wird der Ursachenbegriff unter den PRICL in Abhängigkeit des Ereignisbegriffs definiert. Art. 5.3 PRICL setzt zunächst die Bestimmung eines Ereignisses im Sinne von Art. 5.2 PRICL voraus und erlaubt es von diesem Ereignis aus gesehen auf der Kausalkette auf der Suche nach einer Ursache für die Gefahrverwirklichung bzw. die Haftpflicht begründende Handlung, Unterlassung oder Tatsache weiter zurückzuschreiten. Das Kriterium der „*reasonable foreseeability*“ kontrolliert in der Folge, wie weit auf der Kausalkette zurückgeschritten werden darf.

Art. 5.3 PRICL fasst den Ursachenbegriff nicht neu. Es ist jedoch davon auszugehen, dass z. B. die Tatsache, dass Neuseeland in einem Erdbebengebiet liegt, für die Verwirklichung eines Erdbebens nicht als ursächlich angesehen wird. Auch unter Art. 5.3 PRICL bestehen mehr oder weniger vage Kausalitätserfordernisse. Denn wo genau auf der Kausalkette die Ursache einer Gefahrverwirklichung liegt, kann abstrakt nicht bestimmt werden. Mit dem Kriterium der „*reasonable foreseeability*“ versucht Art. 5.3 PRICL diese Kausalitätserfordernisse zu kontrollieren. Es ist davon auszugehen, dass dieser Mechanismus im Vergleich zur ursachenbasierten Schadenaggregation unter englischem Recht etwas mehr Rechtssicherheit bietet.

Es bleibt abzuwarten, ob Kapitel 5 PRICL in der Praxis für die Schadenaggregation tatsächlich höhere Rechtssicherheit bringen wird oder die Vereinfachung der Aggregationsmechanismen nur theoretisch funktioniert.

## Footnotes

1 Cannawurf und Schwepcke (2013, Rz. 273); Schwepcke (2017, Rz. 155).

2 Vgl. Liebwein (2018, S. 293).

3 Zu den Begriffen „Einzelschadenexzedent“ und „Kumulschadenexzeden“ siehe Katschthaler und Topsch (2017, § 34 Rz. 39).

4 Fausten (2017, S. 1057, 2018, S. 457). Zum Begriff der „Serienschadenklausel“ im Allgemeinen, vgl. Wagner (2017c, Stichwort: „Serienschadenklausel“).

5 Wagner (2017c, Stichwort: „Serienschadenklausel“).

6 Barlow Lyde & Gilbert LLP (2009, Rz. 28.73 ff.); Merkin et al. (2019, Rz. 11–341 ff.); O’Neill et al. (2019, Rz. 7–024). Bemerkenswert ist, dass eine Vielzahl solcher in der englischen Rechtsprechung diskutierter „claims series clauses“ nicht auf eine Serie von Schäden, sondern z. B. auf eine „related series of acts or omissions“, eine „series of related acts or omissions“ oder eine „series of related matters or transactions“ abstellen. Nicht die Einzelschäden sind also Teil einer Serie, sondern die Ursachen oder Umstände der Einzelschäden, siehe z. B. *Lloyd’s TSB General Insurance Holdings v Lloyd’s Bank Group Insurance Co Ltd* [2003] UKHL 48 [12]; *AIG Europe Ltd v Woodman* [2017] UKSC 18 [1]; zum Ganzen William (2020).

7 Fausten (2018, S. 458).

8 Fausten (2017, S. 1062).

9 Dies dürfte nicht zuletzt so sein, weil Rückversicherungsstreitigkeiten in den meisten Fällen unter Ausschluss der Öffentlichkeit vor Schiedsgerichten ausgefochten werden. Vgl. dazu z. B. Noussia (2013, S. 20).

10 Vgl. z. B. Fausten (2017, S. 1062); im Entscheid des schweizerischen Bundesgerichts vom 17. Januar 2014, BGE 140 III 115, E. 6.3, anerkennt das schweizerische Bundesgericht ausdrücklich den hohen Stellenwert der reichen Rechtsprechung englischer Gerichte für die Ausprägung der Rückversicherungspraxis. Jene solle bei der Auslegung und Ergänzung von Rückversicherungsverträgen eine wichtige Rolle spielen.

11 Merkin (2007, S. 380); Barlow Lyde & Gilbert LLP (2009, Rz. 3.1); Looschelders (2013, Rz. 68); Heiss (2018, S. 103, 2020, S. 14); William (2020).

12 Hier ist allerdings beachtlich, dass dieselbe Aggregationsklausel unter englischem Recht wohl anders ausgelegt werden dürfte als unter dem Recht des Bundesstaats New York. Dazu William (2020).

13 Entscheid des schweizerischen Bundesgerichts vom 17. Januar 2014, BGE 140 III 115, E. 6.3.

14 Siehe Fausten (2017, S. 1062 ff.).

15 Cannawurf und Schwepcke (2013, Rz. 274).

16 *Lloyd’s TSB General Insurance Holdings v Lloyd’s Bank Group Insurance Co Ltd* [2003] UKHL 48 [51]; Barlow Lyde & Gilbert LLP (2009, Rz. 4.53); Louw und Tompkinson (1996, S. 11).

17 Vgl. Bork und Wandt (2019, S. 1115).

18 Fausten (2017, S. 1059).

19 Vgl. z. B. Sacher et al. (2020); O’Neill (2020); Melwani und Marland (2020); Mönnich et al. (2020, S. 128 ff.).

20 Tipping et al. (2018).

21 *Axa Reinsurance (UK) Ltd v Field* [1996] 1 WLR 1026 (House of Lords) 1035.

22 William (2020).

201 O’Neill et al. (2019, Rz. 7-013).

23 William (2020).

24 *Equitas Insurance Limited v Municipal Mutual Insurance Limited* [2019] EWCA Civ 718 [91].

25 Barlow Lyde & Gilbert LLP (2009, Rz. 28.1).

26 William (2020).

27 William (2020).

28 William (2020).

29 Näheres dazu bei William (2020).

30 William (2020).

31 *Caudle v Sharp* [1995] CLC 642 (Court of Appeal) 648 (Hervorhebung durch den Verfasser).

32 Dawson’s Field Arbitration, zitiert in: *Kuwait Airways Corp v Kuwait Insurance Co SAK* [1996] 1 Lloyd’s Rep 664 (Commercial Court); *American Centennial Insurance Co v INSCO Ltd* [1996] 1 LRLR 407 (Commercial Court); Barlow Lyde & Gilbert LLP (2009, Rz. 28.6).

33 William (2020).

34 William (2020).

35 Vgl. Dawson’s Field Arbitration zitiert in: *Kuwait Airways Corp v Kuwait Insurance Co SAK* [1996] 1 Lloyd’s Rep 664 (Commercial Court).

36 *Kuwait Airways Corp v Kuwait Insurance Co SAK* [1996] 1 Lloyd’s Rep 664 (Commercial Court).

37 Vgl. Schwepcke und Vetter (2017, Rz. 916); Kiln (1991, S. 194).

38 Im Einzelnen dazu William (2020).

39 Vgl. z. B. *Glencore International AG v Alpina Insurance Co Ltd* [2003] EWHC 2792 (Commercial Court).

40 *Pennsylvania Co for Insurances on Lives and Granting Annuities v Mumford* [1920] 2 KB 537 (Court of Appeal); *Dornoch Ltd v Mauritius Union Assurance Co Ltd (No 2)* [2007] Lloyds Rep IR 350 (Commercial Court).

41 William (2020).

42 *Caudle v Sharp* [1995] CLC 642 (Court of Appeal) 652; vgl. auch *Mann v Lexington Insurance Co* [2000] EWCA Civ 256 (Court of Appeal); Zum Ganzen William (2020).

43 *Phoenix General Insurance Co of Greece SA v Halvanon* [1985] 2 Lloyds Rep 599 (Commercial Court) 612; Edelman und Burns (2013, Rz. 3.63).

44 Statt vieler Cannawurf und Schwepcke (2013, Rz. 93 ff.).

45 Cannawurf und Schwepcke (2013, Rz. 349).

46 *Mabey & Johnson Ltd v Ecclesiastical Insurance Office Plc* [2000] CLC 1570 (Commercial Court) 1573 f.; Barlow Lyde & Gilbert LLP (2009, Rz. 28.40); Birds et al. (2018, Rz. 30–060); Merkin et al. (2019, Rz. 11–321).

47 Cannawurf und Schwepcke (2013, Rz. 349).

48 Cannawurf und Schwepcke (2013, Rz. 350).

49 In *Denby v English and Scottish Maritime Insurance Co Ltd; Yasuda Fire & Marine Co of Europe Ltd v Lloyd’s Underwriting Syndicates no 209, 356* [1998] Lloyds Rep IR 343 (Court of Appeal) werden solche Einzelschäden als ‚unrelated losses‘ bezeichnet; William (2020).

50 *Denby v English and Scottish Maritime Insurance Co Ltd; Yasuda Fire & Marine Co of Europe Ltd v Lloyd’s Underwriting Syndicates no 209, 356* [1998] Lloyds Rep IR 343 (Court of Appeal).

51 Vgl. z. B. *Scott v The Copenhagen Reinsurance Co (UK) Ltd* [2003] EWCA Civ 668 (Court of Appeal) [68]. Zum Ganzen William (2020).

52 Der Ausdruck „linking phrase“ wurde in Anlehnung an *Standard Life Assurance Ltd v Ace European Group* [2012] EWHC 104 (Commercial Court) gewählt, wo der Commercial Court von „description of the link“ spricht. Vgl. auch Clyde & Co (2016), die in diesem Zusammenhang von „connecting words“ sprechen.

53 William (2020); Clyde & Co (2016).

54 Die Begriffe „event“ und „occurrence“ werden i. d. R. synonym verwendet, siehe Dawson’s Field Arbitration, zitiert in: *Kuwait Airways Corp v Kuwait Insurance Co SAK* [1996] 1 Lloyd’s Rep 664 (Commercial Court); *American Centennial Insurance Co v INSCO Ltd* [1996] 1 LRLR 407 (Commercial Court) 413; Barlow Lyde & Gilbert LLP (2009, Rz. 28.6); für einen Überblick über die verschiedenen Bedeutungen des Begriffs „occurrence“ in Aggregationsklauseln siehe William (2020).

55 Für einen Überblick über die englische Rechtsprechung zu den verschiedenen „unifying factors“ siehe William (2020).

56 Im Einzelnen William (2020).

57 Clyde & Co (2016); siehe auch William (2020).

58 Zur zentralen Bedeutung der verwendeten Begriffe siehe *Lloyd’s TSB General Insurance Holdings v Lloyd’s Bank Group Insurance Co Ltd* [2003] UKHL 48 [17].

59 Vgl. z. B. *Caudle v Sharp* [1995] CLC 642 (Court of Appeal) 644.

60 *Caudle v Sharp* [1995] CLC 642 (Court of Appeal) 648 f.

61 Butler und Merkin (Loseblattsammlung, Rz. C–230); William (2020).

62 *Axa Reinsurance (UK) Ltd v Field* [1996] 1 WLR 1026 (House of Lords) 1035.

63 Dawson’s Field Arbitration, zitiert in: *Kuwait Airways Corp v Kuwait Insurance Co SAK* [1996] 1 Lloyd’s Rep 664 (Commercial Court); *Midland Mainline Ltd v Eagle Star Insurance Co Ltd* [2003] EWHC 1771 (Commercial Court) [97]; *Aioi Nissay Dowa Insurance Co Ltd v Heraldglen Ltd and Advent Capital Ltd* [2013] EWHC 154 (Commercial Court) [20].

64 In *Midland Mainline Ltd v Eagle Star Insurance Co Ltd* [2003] EWHC 1771 (Commercial Court) [97] war das Gericht der Ansicht, dass „a decision (…) cannot constitute an event or occurrence“. Demgegenüber befand das Gericht in *Kuwait Airways Corp v Kuwait Insurance Co SAK* [1996] 1 Lloyd’s Rep 664 (Commercial Court), dass die Umsetzung einer Entscheidung ein Ereignis sein könne; in *IRB Brasil Resseguros SA v CX Reinsurance Co Ltd* [2010] EWHC 974 (Commercial Court) [46] wurde dann sogar „the determination of the company to engage in the insulation business and to install Kaylo insulation products“ als Ereignis angesehen.

65 Vgl. z. B. Edelman und Burns (2013, Rz. 4.55), welche die Meinung vertreten, dass Unterlassungen keine Ereignisse sein können. Demgegenüber sind Louw und Tompkinson (1996, S. 10), der vorzugswürdigen Ansicht, dass unter gewissen Voraussetzungen auch Unterlassungen Ereignisse im Sinne einer Aggregationsklausel sein können. Siehe auch *Scott v The Copenhagen Reinsurance Co (UK) Ltd* [2003] EWCA Civ 668 (Court of Appeal) [59]; *Caudle v Sharp* [1995] CLC 642 (Court of Appeal) 649; *Forney v Dominion Insurance Co Ltd* [1969] 1 WLR 928 (Commercial Court) 933.

66 Dawson’s Field Arbitration, zitiert in: *Kuwait Airways Corp v Kuwait Insurance Co SAK* [1996] 1 Lloyd’s Rep 664 (Commercial Court).

67 *Kuwait Airways Corp v Kuwait Insurance Co SAK* [1996] 1 Lloyd’s Rep 664 (Commercial Court); *Aioi Nissay Dowa Insurance Co Ltd v Heraldglen Ltd and Advent Capital Ltd* [2013] EWHC 154 (Commercial Court) [31].

68 *Caudle v Sharp* [1995] CLC 642 (Court of Appeal) 648.

69 Vgl. *Scott v The Copenhagen Reinsurance Co (UK) Ltd* [2003] EWCA Civ 668 (Court of Appeal) [65].

70 Dawson’s Field Arbitration, zitiert in: *Scott v The Copenhagen Reinsurance Co (UK) Ltd* [2003] EWCA Civ 668 (Court of Appeal) [52]; *Caudle v Sharp* [1995] CLC 642 (Court of Appeal) 648; *Scott v The Copenhagen Reinsurance Co (UK) Ltd* [2003] EWCA Civ 668 (Court of Appeal) [65]; Dazu im Ganzen William (2020).

71 *Caudle v Sharp* [1995] CLC 642 (Court of Appeal) 648.

72 *Scott v The Copenhagen Reinsurance Co (UK) Ltd* [2003] EWCA Civ 668 (Court of Appeal) [63].

73 *Scott v The Copenhagen Reinsurance Co (UK) Ltd* [2003] EWCA Civ 668 (Court of Appeal) [68].

74 *Scott v The Copenhagen Reinsurance Co (UK) Ltd* [2003] EWCA Civ 668 (Court of Appeal) [68].

75 Vgl. *Scott v The Copenhagen Reinsurance Co (UK) Ltd* [2003] EWCA Civ 668 (Court of Appeal) [81]; *MIC Simmonds v Gammell* [2016] EWHC 2515 (Commercial Court) [36, 39].

76 *Scott v The Copenhagen Reinsurance Co (UK) Ltd* [2003] EWCA Civ 668 (Court of Appeal) [81].

77 *Scott v The Copenhagen Reinsurance Co (UK) Ltd* [2003] EWCA Civ 668 (Court of Appeal) [81].

78 Dawson’s Field Arbitration, zitiert in: *Kuwait Airways Corp v Kuwait Insurance Co SAK* [1996] 1 Lloyd’s Rep 664 (Commercial Court).

79 *Scott v The Copenhagen Reinsurance Co (UK) Ltd* [2003] EWCA Civ 668 (Court of Appeal) [68]; *Caudle v Sharp* [1995] CLC 642 (Court of Appeal) 648 f.

80 *Caudle v Sharp* [1995] CLC 642 (Court of Appeal) 648 f.; *Scott v The Copenhagen Reinsurance Co (UK) Ltd* [2003] EWCA Civ 668 (Court of Appeal) [63].

81 *Kuwait Airways Corp v Kuwait Insurance Co SAK* [1996] 1 Lloyd’s Rep 664 (Commercial Court).

82 *Aioi Nissay Dowa Insurance Co Ltd v Heraldglen Ltd and Advent Capital Ltd* [2013] EWHC 154 (Commercial Court) [21].

83 Dawson’s Field Arbitration, zitiert in: *Kuwait Airways Corp v Kuwait Insurance Co SAK* [1996] 1 Lloyd’s Rep 664 (Commercial Court).

84 Dawson’s Field Arbitration, zitiert in: *Kuwait Airways Corp v Kuwait Insurance Co SAK* [1996] 1 Lloyd’s Rep 664 (Commercial Court); vgl. auch *Aioi Nissay Dowa Insurance Co Ltd v Heraldglen Ltd and Advent Capital Ltd* [2013] EWHC 154 (Commercial Court) [23].

85 *Mann v Lexington Insurance Co* [2000] EWCA Civ 256 (Court of Appeal) 1423.

86 *Midland Mainline Ltd v Eagle Star Insurance Co Ltd* [2003] EWHC 1771 (Commercial Court) [99].

87 *Aioi Nissay Dowa Insurance Co Ltd v Heraldglen Ltd and Advent Capital Ltd* [2013] EWHC 154 (Commercial Court) [38].

88 Dawson’s Field Arbitration, zitiert in: *Kuwait Airways Corp v Kuwait Insurance Co SAK* [1996] 1 Lloyd’s Rep 664 (Commercial Court).

89 In *IRB Brasil Resseguros SA v CX Reinsurance Co Ltd* [2010] EWHC 974 (Commercial Court) [46]. In seinem Entscheid erwänte der Commercial Court die *unity of location* nicht ausdrücklich. Er erklärte lediglich, der *unities test* sei erfüllt gewesen.

90 *Kuwait Airways Corp v Kuwait Insurance Co SAK* [1996] 1 Lloyd’s Rep 664 (Commercial Court); *Aioi Nissay Dowa Insurance Co Ltd v Heraldglen Ltd and Advent Capital Ltd* [2013] EWHC 154 (Commercial Court) [22].

91 William (2020).

92 Dawson’s Field Arbitration, zitiert in: *Kuwait Airways Corp v Kuwait Insurance Co SAK* [1996] 1 Lloyd’s Rep 664 (Commercial Court).

93 *Aioi Nissay Dowa Insurance Co Ltd v Heraldglen Ltd and Advent Capital Ltd* [2013] EWHC 154 (Commercial Court) [22].

94 Zum Ganzen William (2020).

95 Dawson’s Field Arbitration, zitiert in: *Kuwait Airways Corp v Kuwait Insurance Co SAK* [1996] 1 Lloyd’s Rep 664 (Commercial Court).

96 William (2020).

97 *Kuwait Airways Corp v Kuwait Insurance Co SAK* [1996] 1 Lloyd’s Rep 664 (Commercial Court).

98 Dawson’s Field Arbitration, zitiert in: *Kuwait Airways Corp v Kuwait Insurance Co SAK* [1996] 1 Lloyd’s Rep 664 (Commercial Court). Demgegenüber entschied das Schiedsgericht in *Aioi Nissay Dowa Insurance Co Ltd v Heraldglen Ltd and Advent Capital Ltd* [2013] EWHC 154 (Commercial Court) [20], die gemeinsame Absicht aller Attentäter, am 11. September 2001 auf das World Trade Center ein Attentat zu verüben, begründe keine *unity of intent*.

99 *Midland Mainline Ltd v Eagle Star Insurance Co Ltd* [2003] EWHC 1771 (Commercial Court) [100].

100 Dazu William (2020).

101 William (2020).

102 *MIC Simmonds v Gammell* [2016] EWHC 2515 (Commercial Court) [29].

103 *MIC Simmonds v Gammell* [2016] EWHC 2515 (Commercial Court) [29].

104 *Aioi Nissay Dowa Insurance Co Ltd v Heraldglen Ltd and Advent Capital Ltd* [2013] EWHC 154 (Commercial Court) [37].

105 Mit Lord Justice Evans Worten darf das Ereignis in der Kausalkette von den Einzelschäden nicht zu weit entfernt sein (*not be too remote*). Siehe *Caudle v Sharp* [1995] CLC 642 (Court of Appeal) 648.

106 Barlow Lyde & Gilbert LLP (2009, Rz. 28.38, 28.40).

107 So geschehen z. B. in *Municipal Mutual Insurance Ltd v Sea Insurance Co Ltd* [1998] CLC 957 (Court of Appeal) 961.

108 Z. B. *Cox v Bankside Members Agency Ltd* [1995] CLC 671 (Court of Appeal) 679; *Standard Life Assurance Ltd v Oak Dedicated Ltd* [2008] EWHC 222 (Commercial Court) [14]; *Standard Life Assurance Ltd v Ace European Group* [2012] EWHC 104 (Commercial Court) [225].

109 Z. B. *Municipal Mutual Insurance Ltd v Sea Insurance Co Ltd* [1998] CLC 957 (Court of Appeal) 961; *Countrywide Assured Group Plc v Marshall* [2002] EWHC 2082 (Commercial Court) [3].

110 Barlow Lyde & Gilbert LLP (2009, Rz. 28.49); Edelman und Burns (2013, Rz. 4.59 ff.); Merkin et al. (2019, Rz. 11–335); *Municipal Mutual Insurance Ltd v Sea Insurance Co Ltd* [1998] CLC 957 (Court of Appeal) 967; vgl. auch *Countrywide Assured Group Plc v Marshall* [2002] EWHC 2082 (Commercial Court) [15].

111 Z. B. *Municipal Mutual Insurance Ltd v Sea Insurance Co Ltd* [1998] CLC 957 (Court of Appeal) 966; *Countrywide Assured Group Plc v Marshall* [2002] EWHC 2082 (Commercial Court) [3]; *Tokio Marine Europe Insurance Ltd v Novae Corporate Underwriting Ltd* [2014] EWHC 2105 (Commercial Court) [8].

112 *Cox v Bankside Members Agency Ltd* [1995] CLC 180 (Commercial Court) 679.

113 *Axa Reinsurance (UK) Ltd v Field* [1996] 1 WLR 1026 (House of Lords) 1035.

114 *Countrywide Assured Group Plc v Marshall* [2002] EWHC 2082 (Commercial Court) [15]; vgl. auch Merkin (2012, S. 145).

115 *Brown (RE) v GIO Insurance Ltd* [1998] Lloyds Rep IR 201 (Court of Appeal) 204.

116 William (2020).

117 Merkin (2012, S. 146 f.).

118 Merkin (2012, S. 146).

119 Merkin (2012, S. 145).

120 *Axa Reinsurance (UK) Ltd v Field* [1996] 1 WLR 1026 (House of Lords) 1035.

121 *American Centennial Insurance Co v INSCO Ltd* [1996] 1 LRLR 407 (Commercial Court) 414. Konkret erklärte der Commercial Court, „there must also be a limit to the degree of remoteness that is acceptable“.

122 Louw und Tompkinson (1996, S. 8).

123 *Countrywide Assured Group Plc v Marshall* [2002] EWHC 2082 (Commercial Court) [5].

124 *American Centennial Insurance Co v INSCO Ltd* [1996] 1 LRLR 407 (Commercial Court) 414.

125 *Municipal Mutual Insurance Ltd v Sea Insurance Co Ltd* [1998] CLC 957 (Court of Appeal) 967.

126 Merkin (2012, S. 145).

127 Vgl. *Axa Reinsurance (UK) Ltd v Field* [1996] 1 WLR 1026 (House of Lords) 1035.

128 Hierin könnte gegebenenfalls eine Unterlassung der Informationsbeschaffung/Risikoevaluierung durch den Underwriter gesehen werden. Zwischen dieser Unterlassung und den Einzelschäden kann unter Umständen eine hypothetische Kausalität angenommen werden.

202 Hier ist jedenfalls auch keine hypothetische Kausalität ersichtlich.

129 William (2020).

130 Vgl. Heiss (2020, S. 8 ff.); Cerini (2020, S. 26); Bork und Wandt (2019, S. 1115); Heiss (2018, S. 95 ff.).

131 Inhaber des Lehrstuhls für Privatrecht, Rechtsvergleichung und Internationales Privatrecht an der Universität Zürich.

132 Stellvertretender Vorstand des Instituts für Zivilrecht an der Universität Wien.

133 Geschäftsführender Direktor des Instituts für Versicherungsrecht an der Goethe Universität Frankfurt a. M.

134 Sie bilden zusammen das sogenannte Principles Drafting Commitee (PDC). Die Mitglieder des PDC sind ersichtlich unter: <https://www.ius.uzh.ch/de/research/projects/pricl/whoweare/draftingcommittee.html>, zuletzt besucht am 19. August 2020.

135 Die Zusammensetzung der sog. Advisory Groups wird aus der Projektwebsite ersichtlich: <https://www.ius.uzh.ch/de/research/projects/pricl/whoweare.html>, zuletzt besucht am 14. August 2020.

136 Bork und Wandt (2019, S. 1116); Heiss (2018, S. 101 ff.).

137 Heiss (2020, S. 5 f.).

138 Die PRICL stehen zum freien Download bereit unter: <https://www.ius.uzh.ch/de/research/projects/pricl.html>, zuletzt besucht am 14. August 2020.

139 Kommentar C6 zu Art. 5.1 PRICL.

140 Vgl. Kommentar C4 zu Art. 5.1 PRICL; William (2020).

141 Siehe auch Kommentar C4 zu Art. 5.2 PRICL; William (2020).

142 Für die Beurteilungskriterien von Lord Justice Evans, siehe *Caudle v Sharp* [1995] CLC 642 (Court of Appeal) 648.

143 William (2020).

144 Dazu Kommentare C21 ff. zu Art. 5.1 PRICL.

145 Gemäss Kommentare C30 ff. zu Art. 5.2 PRICL werden Lebensversicherungspolicen ebenfalls unter Absatz 1 von Art. 5.2 PRICL behandelt.

146 Vgl. Wagner (2017b, Stichwort: „Sachversicherung“); Baumunk (2003, S. 337).

147 Auch für den Fall, dass der Rückversicherungsvertrag nicht ausdrücklich vorsieht, gegen welche Gefahren er Schutz bietet (all-risk Rückversicherungspolicen), kann Art. 5.2 (1) PRICL zur Anwendung gebracht werden. Siehe dazu Kommentar C11 zu Art. 5.2 PRICL.

148 William (2020).

149 Siehe Kommentar C9 zu Art. 5.2 PRICL.

150 Siehe Kommentar C10 zu Art. 5.2 PRICL; William (2020).

151 Kommentar C10 zu Art. 5.2 PRICL.

152 Vgl. Wagner (2017a, Stichwort: „Haftpflichtversicherung“); Baumunk (2003, S. 332).

153 Vgl. Butler und Merkin (Loseblattsammlung, Rz. C–0221).

154 William (2020). Damit im Einklang dürfte auch *Caudle v Sharp* [1995] CLC 642 (Court of Appeal) 649, stehen. Demgegenüber wurde in *IRB Brasil Resseguros SA v CX Reinsurance Co Ltd* [2010] EWHC 974 (Commercial Court) [46] offenbar ein anderer Ansatz verfolgt.

155 Kommentar C13 zu Art. 5.2 PRICL.

156 Kommentar C13 zu Art. 5.2 PRICL.

157 Kommentar C14 zu Art. 5.2 PRICL.

158 Kommentar C4 zu Art. 5.2 PRICL.

159 Kommentar C18 zu Art. 5.2 PRICL.

160 Kommentar C20 zu Art. 5.2 PRICL.

161 Kommentar C21 zu Art. 5.2 PRICL.

162 Kommentar C22 zu Art. 5.2 PRICL.

163 Kommentar C22 zu Art. 5.2 PRICL.

164 Kommentar C19 zu Art. 5.2 PRICL.

165 Zu den Gründen dafür, siehe oben, Abschn. 7.2.1.

166 Kommentar C2 zu Art. 5.3 PRICL.

167 Illustration I1 zu Art. 5.2 PRICL.

168 Kommentar C7 zu Art. 5.3 PRICL.

169 Kommentar C8 zu Art. 5.3 PRICL.

170 Wie bereits erwähnt, können alle Ereignisse auch als Ursachen bezeichnet werden. Vgl. oben Abschn. 6.

171 Kommentar C8 zu Art. 5.3 PRICL.

172 Kommentar C9 zu Art. 5.3 PRICL.

173 Kommentar C9 zu Art. 5.3 PRICL.

174 Kommentar C12 zu Art. 5.3 PRICL.

175 Kommentar C13 zu Art. 5.3 PRICL.

176 Kommentar C19 zu Art. 5.3 PRICL.

177 Vgl. Kommentar C19 zu Art. 5.3 PRICL.

178 *Scott v The Copenhagen Reinsurance Co (UK) Ltd *[2003] EWCA Civ 668 (Court of Appeal) [68].

203 Dazu Kommentar C11 zu Art. 5.2 PRICL.
